# Knowledge Distillation Meets Reinforcement Learning: A Cluster-Driven Approach to Image Processing

**DOI:** 10.3390/s26010209

**Published:** 2025-12-28

**Authors:** Titinunt Kitrungrotsakul, Yingying Xu, Preeyanuch Srichola

**Affiliations:** 1 Research Center for Space Computing System, Zhejiang Lab, Hangzhou 311121, China; cs_ying@zhejianglab.org; 2 Kasetsart Agricultural and Agro-Industrial Product Improvement Institute, Kasetsart University, Bangkok 10900, Thailand; aappua@ku.ac.th; 3 Cellulose for Future Materials and Technologies Special Research Unit, Department of Biotechnology, Faculty of Agro-Industry, Kasetsart University, Bangkok 10900, Thailand

**Keywords:** remote sensing, knowledge distillation, reinforcement learning, classification, retrieval

## Abstract

Knowledge distillation (KD) enables the training of lightweight yet effective models, particularly in the visual domain. Meanwhile, reinforcement learning (RL) facilitates adaptive learning through environment-driven interactions, addressing the limitations of KD in handling dynamic and complex tasks. We propose a novel two-stage framework integrating Knowledge Distillation with Reinforcement Learning (KDRL) to enhance model adaptability to complex data distributions, such as remote sensing and medical imaging. In the first stage, supervised fine-tuning guides the student model using logit and feature-based distillation. The second stage refines the model via RL, leveraging confidence-based and cluster alignment rewards while dynamically reducing reliance on task loss. By combining the strengths of supervised knowledge distillation and reinforcement learning, KDRL provides a comprehensive approach to address the dual challenges of model efficiency and domain heterogeneity. A key innovation is the introduction of auxiliary layers within the student encoder to evaluate and reward the alignment of the characteristics with the teacher’s cluster centers, promoting robust feature learning. Our framework demonstrates superior performance and computational efficiency across diverse tasks, establishing a scalable design for efficient model training. Across remote sensing benchmarks, KDRL boosts the lightweight CLIP/ViT-B-32 student to 69.51% zero-shot accuracy on AID and 80.08% on RESISC45; achieves state-of-the-art cross-modal retrieval on RSITMD with 67.44% (I→T) and 74.76% (T→I) at R@10; and improves DIOR-RSVG visual-grounding precision to 64.21% at Pr@0.9. These gains matter in real deployments by reducing missed targets and speeding analyst search on resource-constrained platforms.

## 1. Introduction

Knowledge distillation (KD) [1,2,3] has emerged as a powerful technique for transferring knowledge from large, high-capacity teacher models to compact, efficient student models. By leveraging soft target distributions and feature-based guidance, KD enables smaller models to achieve competitive performance while reducing computational costs. This paradigm is particularly valuable for resource-constrained environments, where deploying large-scale models is impractical.

Deep learning models have been increasingly applied to complex domains, where high-dimensional, multimodal data pose significant challenges. Remote sensing tasks—such as land cover classification, object detection, and image retrieval—require models capable of handling diverse spatial and spectral features [4,5,6]. Similarly, medical imaging involves high-stakes decision-making, privacy constraints, and substantial variations in data quality across institutions [7,8,9]. In both domains, knowledge transfer from high-performing teacher models can improve generalization and robustness, making KD a promising solution.

However, traditional KD methods have notable limitations when applied to these complex domains. Conventional KD operates with fixed objectives, assuming that the knowledge transfer process is uniform across all instances. This assumption does not hold for remote sensing and medical imaging, where data heterogeneity, domain shifts, and task complexity significantly affect knowledge transfer effectiveness. For example, in remote sensing, satellite images vary based on geographical regions, acquisition conditions, and sensor types, making static KD objectives suboptimal. In medical imaging, factors such as inter-patient variability and data imbalance can degrade the effectiveness of standard KD strategies.

Scientific problem: In practice, remote sensing and medical imaging pipelines often require lightweight models that run under strict latency and memory budgets, yet must remain reliable under domain shift (e.g., seasonal changes, sensor differences, and heterogeneous scene layouts). The central problem we address is how to transfer the generalization and robust representations of a large vision–language teacher into a compact student without over-regularizing the student or amplifying teacher biases.

Literature gap: Most KD approaches for vision tasks rely on static objectives (logit/feature matching) with fixed weights applied uniformly across samples and tasks, while RL-based KD studies typically embed RL inside the KD loop to adjust distillation signals, rather than using RL to refine a trained student using task-aware rewards. This leaves a gap: an objective mechanism to adapt the student after KD toward downstream task criteria, while keeping inference efficient.

These challenges highlight the need for an adaptive knowledge transfer mechanism that dynamically refines student models based on task-specific requirements. Relying solely on KD can lead to suboptimal feature representations and degraded performance in cases where the student model struggles to generalize across complex data distributions.

Reinforcement learning (RL) offers an alternative approach by enabling models to learn adaptive policies through interaction with an environment [10,11,12]. Recent advances, such as REFT [13], have demonstrated the potential of RL in fine-tuning pre-trained models for specific tasks. While these approaches optimize model performance by adapting learning strategies dynamically, their application to KD remains underexplored. Integrating RL into KD offers a promising direction for overcoming the limitations of traditional knowledge transfer, allowing student models to refine their learning process based on reinforcement-driven feedback.

To address these challenges, we introduce a novel two-stage framework that integrates KD with RL to refine student models through reward-driven optimization. The first stage employs supervised learning with KD, ensuring alignment between the student and teacher models. The second stage introduces reinforcement learning to dynamically refine knowledge transfer, based on our carefully designed reward mechanisms, including cluster-based rewards and confidence-based rewards. During the fine-tuning process, dynamic KD-RL adjustment is employed to assign varying levels of importance to KD and RL.

By addressing the fundamental challenges of KD through reinforcement-driven optimization, our research establishes a more adaptive and scalable knowledge transfer paradigm, significantly improving performance in complex real-world applications. Our work makes the following key contributions:

- Integration of KD and RL: We propose the first comprehensive framework that unifies KD with RL for adaptive student model refinement.
- Dynamic and Reward-Driven Training: Our multi-reward RL mechanism enables task-specific optimization beyond static KD.
- Scalable and Generalizable Framework: The method is designed to handle diverse domains such as remote sensing and medical imaging.

## 2. Related Work

This section reviews knowledge distillation and reinforcement learning studies most relevant to lightweight vision models and highlights the literature gap addressed by KDRL. Most KD approaches for vision tasks optimize fixed combinations of logit and feature matching losses; their weights are typically static and applied uniformly across samples. Meanwhile, KD + RL hybrids mainly use RL to select teachers, choose distillation routes, or adjust distillation strength within the KD loop. In contrast, KDRL adopts a two-stage strategy: a strong KD initialization is followed by reward-driven refinement that directly optimizes task-aware criteria (cluster alignment and confidence), while keeping inference unchanged.

### 2.1. Knowledge Distillation

KD has been extensively studied as a model compression technique. Hinton [1] first introduced the foundational concept of distilling knowledge from a large teacher model to a smaller student model by matching logits. Recent advancements, such as feature-based KD and attention transfer, have expanded the applicability of KD to tasks like object detection [14,15] and semantic segmentation [16,17]. In addition, advanced specific KD strategies/techniques have also been proposed for specific domains [18,19] or solving specific problems [20]. For example, TAKD [21] proposes a target-aware KD method to create a lightweight remote sensing scene classification model, effectively distilling target-specific knowledge from RS images, which often exhibit large variations in object scales. RoS-KD [22] introduces a robust stochastic KD framework to incorporate additional smoothening in the distillation step for noisy medical imaging, where knowledge is distilled from multiple teacher models to handle noisy labels effectively. The impressive results highlight the promising potential of KD in specialized domains such as remote sensing and medical imaging.

### 2.2. Reinforcement Learning

RL has shown promise in tasks requiring sequential decision making, such as object tracking, robotic manipulation, and games [23]. With the rapid development of large foundation models, some research has focused on leveraging these pre-trained foundation models to generate dense rewards for RL tasks [24,25]. Recently, the application of RL has expanded into fine-tuning the pre-trained models. A prominent example is REFT [13], which utilizes RL to fine-tune pre-trained models for specific tasks, effectively guiding the model to adapt to task-specific challenges. Another work [26] proposes to use RL techniques that align models with a task reward to tune vision models, addressing the issue of misalignment between model predictions and the task risk. In contrast to the work that applies RL in the fine-tuning phase, our approach integrates RL with KD, where we provide dynamic rewards based on the alignment of student features with teacher cluster centers.

### 2.3. Integration of Knowledge Distillation and Reinforcement Learning

Several recent studies have explored combining KD with RL to mitigate the limitations of static distillation schedules and weights. For example, Lee et al. (2021) [27] explicitly introduce RL into the distillation process for vision tasks, using reinforcement-driven signals to stimulate a student’s ability to absorb teacher knowledge beyond fixed hyperparameter settings. RLKD [28] further extends this direction by leveraging RL signals to guide instance-level distillation decisions.

These approaches are conceptually close in that RL is used to control the KD procedure (e.g., how/when to distill, or which distillation signals to emphasize). In contrast, KDRL adopts a two-stage design: Stage 1 performs standard supervised KD to obtain a stable student initialization, while Stage 2 performs a separate reward-driven refinement with rewards derived from the teacher’s representation geometry (cluster centers) and prediction confidence. This decoupling avoids embedding an RL controller directly into the KD loop, and it is particularly practical for remote sensing settings where the teacher is fixed and reward computation can be implemented as a lightweight auxiliary objective.

Overall, prior KD + RL studies motivate the use of adaptive reinforcement signals during distillation; our contribution is to instantiate these signals as cluster- and confidence-based rewards and to integrate them into a two-stage pipeline tailored to lightweight student models in remote sensing and medical imaging.

## 3. Methodology

### 3.1. Problem Definition

Deep learning models have demonstrated remarkable performance across various domains. However, deploying these models in real-world applications is challenging due to their high computational demands, memory consumption, and limited scalability. Domains such as remote sensing and medical imaging exacerbate these challenges due to unique characteristics: remote sensing data involves diverse spatial resolutions and temporal variations, while medical imaging data exhibits significant heterogeneity from variations in modalities (e.g., CT and MRI) and patient demographics.

To address these issues, KD has emerged as a promising technique. In KD, a large pre-trained teacher model *T* transfers its knowledge to a smaller student model *S*, which is designed to be lightweight and efficient. This process typically involves minimizing the difference between the teacher and student outputs using the following objectives:

- *Logit Matching:* The student’s output logits are aligned with the teacher’s, enabling *S* to emulate *T*’s decision boundaries.
- *Feature Alignment:* Intermediate feature representations of *S* are aligned with *T*, facilitating better transfer of structural information.
- *Task-Specific Supervision:* A task loss (e.g., classification or detection) ensures that *S* learns task-relevant features.

Despite its success, traditional KD methods often struggle when the student capacity is very small or when the downstream data distribution deviates from the teacher’s pre-training domain. To better adapt the student beyond static KD, we introduce an RL-inspired formulation for the refinement stage. Concretely, we treat each mini-batch update as a one-step contextual bandit: the student produces intermediate representations (state), a lightweight refinement module (RLayer) produces an additive residual (action), and a composite reward guides the refinement.

- State: the intermediate representations extracted by the student model (e.g., transformer block outputs).
- Action: an additive refinement produced by RLayer, applied to the student representation (Section 3.4).
- Reward: a composite score that encourages (i) alignment to the teacher representation geometry (cluster-based reward) and (ii) confident predictions (confidence-based reward).

Accordingly, we maximize the expected reward and minimize its negation:

$$L_{\mathrm{RL}}=-E_{x∼D}\left[r(x)\right],\;r\left(x\right)=\delta \;r_{\mathrm{cluster}}\left(x\right)+\eta \;r_{\mathrm{conf}}\left(x\right),$$

where δ and η balance the reward components. Note that this refinement is deterministic and differentiable, so we optimize $L_{\mathrm{RL}}$ directly with standard backpropagation and do not require policy gradients or an explicit actor–critic.

The final training objective combines KD and reward-driven refinement:

$$L_{\mathrm{final}}=w\left(t\right)\;L_{\mathrm{KD}}+\left(1-w(t)\right)\;L_{\mathrm{RL}},$$

where w(t) is a time-dependent weight (defined in Section 3.4) that decays from KD to refinement.

This problem setting emphasizes the need for a unified framework that integrates KD and RL to handle the challenges of heterogeneous data distributions in remote sensing and medical imaging.

### 3.2. Knowledge Distillation with Reinforcement Learning (KDRL)

In terms of a framework overview, KDRL consists of two sequential stages operating on a frozen teacher *T* and a lightweight student *S*: (i) *KD initialization*, where *S* learns from *T* using logit and feature matching plus task loss; and (ii) *reward-driven refinement*, where small *refinement layers* (RLayer) update intermediate student representations to better satisfy task-aware rewards. The framework depends on (a) a pre-trained vision–language teacher (e.g., RemoteCLIP) to provide high-quality embeddings and logits, and (b) teacher feature clusters computed once (offline) to define a stable cluster-alignment reward.

**Main components.**

- **Teacher T (frozen):** provides logits and embedding features, used to compute cluster centers.
- **Student S:** the compact image encoder to be deployed at inference time.
- **RLayer (train-time only):** lightweight MLP/residual adapters inserted after selected student blocks to produce refinement actions.
- **Reward modules:** a cluster-alignment reward (matching teacher cluster assignments/centroids) and a confidence reward (encouraging confident, teacher-consistent predictions).
- **Dynamic schedule:** gradually decays KD weight and increases reward-driven emphasis to stabilize optimization.

**General flow.** Compute teacher cluster centers $\left\{\mu_{k}\right\}$ offline; train *S* with KD (Stage 1); then enable RLayer and optimize the Stage 2 objective with dynamic KD–RL weighting; finally, discard RLayer for deployment so the inference cost matches the student backbone.

**Figure 1:**
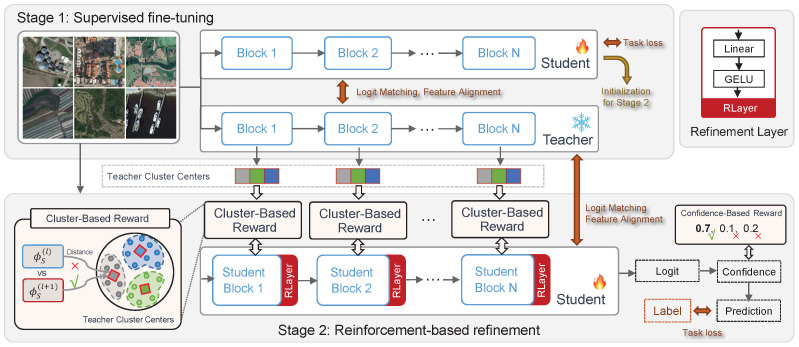
Overview of our Knowledge Distillation with Reinforcement Learning (KDRL) framework. KDRL operates in two stages: Stage 1 (**top**) involves supervised fine-tuning, where the model is initialized using knowledge distillation (KD) and task-specific loss to establish a robust baseline. Stage 2 (**bottom**) incorporates reinforcement learning (RL) for further refinement, leveraging RL-based losses such as cluster loss and confidence loss to enhance the model’s performance and generalizability. This two-stage process ensures an effective balance between initial knowledge transfer and adaptive optimization. Teacher cluster centers are computed offline by clustering teacher embeddings and are used as fixed anchors during Stage 2 to compute the cluster-based reward (as indicated by the arrows).

To address the challenges identified in the previous section, we propose Knowledge Distillation with Reinforcement Learning (KDRL), a novel framework designed to enhance model efficiency and adaptability in domains with complex and heterogeneous data distributions. An overview of KDRL is illustrated in Figure 1. The KDRL framework is systematically divided into two distinct training stages: (1) supervised fine-tuning with knowledge distillation and (2) reinforcement-based refinement.

The supervised fine-tuning with knowledge distillation stage focuses on training an initial student model under the guidance of a pre-trained teacher model. This stage leverages classical KD techniques to align the student model’s outputs and intermediate feature representations with those of the teacher. The objective is to enable the student model to capture essential task-relevant information efficiently, thereby building a solid foundation for subsequent refinement.

The reinforcement-based refinement stage aims to further optimize the student model by incorporating task-specific adaptability and domain-aware decision-making. In this stage, the student model is treated as an agent within a RL framework, where the state is defined by the model’s learned representations, actions correspond to parameter updates, and the reward function provides feedback based on task-specific criteria, such as domain alignment, or other custom metrics. This stage allows the model to dynamically refine its parameters, enhancing performance on diverse and unseen data distributions.

By combining the strengths of supervised knowledge distillation and reinforcement learning, KDRL provides a comprehensive approach to address the dual challenges of model efficiency and domain heterogeneity. The two stages are seamlessly integrated, with the KD phase laying a strong groundwork for the RL stage, and the refinement phase adding adaptive capabilities to the student model. This ensures that the final model not only achieves high task performance but also generalizes effectively across various data domains.

### 3.3. Stage 1: Supervised Fine-Tuning with Knowledge Distillation

The first stage of our framework focuses on initializing the student model *S* using KD from the teacher model *T*. This step ensures that *S* effectively captures the essential knowledge from *T* and establishes a robust foundation for downstream tasks.

**Logit Matching:** The logit outputs of the teacher and student are aligned using Kullback–Leibler (KL) divergence:

(1) $$L_{\mathrm{KD},\mathrm{logits}}=\mathrm{KL}\left(\sigma (T(x)/\tau )\|\sigma (S(x)/\tau )\right);$$

where σ denotes the softmax function, and τ is the temperature parameter controlling the smoothness of the output distribution.

**Feature Alignment:** The intermediate features extracted by the teacher ($\phi_{T}$) and student ($\phi_{S}$) are aligned using a mean squared error (MSE) loss:

(2) $$L_{\mathrm{KD},\mathrm{features}}={∥\phi_{T}\left(x\right)-\phi_{S}\left(x\right)∥}_{2}^{2};$$

**Task-Specific Loss:** The student is trained to optimize a supervised task loss (e.g., cross-entropy for classification):

(3) $$L_{\mathrm{task}}=\frac{1}{N}\sum_{i=1}^{N}\ell \left(f_{S}\left(x_{i}\right),y_{i}\right);$$

where $f_{S}\left(x_{i}\right)$ is the student’s prediction for input $x_{i}$, and $y_{i}$ is the ground truth label.

The overall objective in the KD stage is a weighted combination of these losses:

(4) $$L_{\mathrm{KD}}=\alpha L_{\mathrm{KD},\mathrm{logits}}+\beta L_{\mathrm{KD},\mathrm{features}}+\gamma L_{\mathrm{task}};$$

where α,β,γ are hyperparameters. Unless otherwise stated, we tune α,β,γ on a held-out validation split using a small grid search {0.25,0.5,0.75,1.0,1.25,1.5} and use the default setting α=0.75, β=0.75, and γ=1.0 across datasets, which we found to be stable and competitive.

The overall training objective for this stage is a weighted combination of these losses, balancing knowledge transfer and task-specific learning.

### 3.4. Stage 2: Reinforcement-Based Refinement

The second stage refines the student model *S* beyond static KD by optimizing a reward-shaped objective. As described in the problem definition, we treat each mini-batch update as a one-step contextual bandit: the student produces intermediate representations (state), a lightweight refinement layer produces an additive residual (action), and a composite reward guides the refinement.

#### 3.4.1. Refinement Layer (RLayer)

RLayer is a lightweight two-layer MLP (Linear–GELU–Linear) applied to the output of a student block. Let $h_{\ell }$ denote the representation at block *ℓ*. RLayer produces an action $a_{\ell }=g_{\psi }\left(h_{\ell }\right)$, and the refined representation is computed via a residual connection:

(5) $${\tilde{h}}_{\ell }=h_{\ell }+a_{\ell }.$$

In practice, we attach RLayer to the last few student blocks (Figure 1) and fine-tune the student jointly with these refinement parameters ψ during Stage 2.

#### 3.4.2. Cluster-Based Reward

To encourage the student representation to preserve the teacher’s geometric structure, we first compute teacher cluster centers ${\left\{c_{k}\right\}}_{k=1}^{K}$ offline by applying *k*-means on teacher embeddings. During Stage 2, each refined student embedding $\tilde{z}$ is assigned to its nearest teacher center $c_{k^{*}}$ (cosine distance), and we define the cluster-based reward as cosine similarity:

(6) $$r_{\mathrm{cluster}}\left(x\right)=\cos\left(\tilde{z}\left(x\right),c_{k^{*}}\left(x\right)\right).$$

We optimize this reward by minimizing $L_{\mathrm{cluster}}=-r_{\mathrm{cluster}}$.

#### 3.4.3. Confidence-Based Reward

We further encourage confident predictions that remain consistent with the teacher. Let $p_{T}\left(x\right)=\sigma \left(T\left(x\right)/\tau \right)$ and $p_{S}\left(x\right)=\sigma \left(S\left(x\right)/\tau \right)$ be teacher and student probabilities (temperature τ). We define the teacher pseudo-label ${\hat{y}}_{T}\left(x\right)=\arg\max_{y}p_{T}^{y}\left(x\right)$ and compute:

(7) $$r_{\mathrm{conf}}\left(x\right)=p_{S}^{{\hat{y}}_{T}\left(x\right)}\left(x\right).$$

Similarly, we optimize this term by minimizing $L_{\mathrm{conf}}=-r_{\mathrm{conf}}$.

#### 3.4.4. Stage-2 Objective and Dynamic KD–RL Schedule

The Stage 2 refinement loss combines task supervision with the two reward-shaped terms:

(8) $$L_{\mathrm{ref}}=L_{\mathrm{task}}+\delta \;L_{\mathrm{cluster}}+\eta \;L_{\mathrm{conf}},$$

where δ and η balance the cluster and confidence components. We then blend Stage 1 KD and Stage 2 refinement with a decaying weight

(9) w(t)=exp(−λt),

and optimize

(10) $$L_{\mathrm{final}}=w\left(t\right)\;L_{\mathrm{KD}}+\left(1-w(t)\right)\;L_{\mathrm{ref}}.$$

**Hyperparameters.** We tune δ,η∈{0.1,0.3,0.5,0.7,1.0} and λ∈{0.01,0.03,0.05,0.1} on a validation split and report the best configuration. Unless otherwise stated, we use δ=0.7, η=0.3, and λ=0.05 as defaults, which were stable across the benchmarks. Stage 2 is run for a small number of refinement epochs (5 by default), so the additional training overhead is modest (Section 4.2).

## 4. Experimental Results

### 4.1. Implementation Details and Setup

To evaluate the performance of the proposed KDRL framework, we conduct two comprehensive experiments: (1) zero-shot image classification, and (2) image–text retrieval. These experiments are designed to systematically assess the effectiveness of KDRL across diverse tasks and scenarios.

For all tasks, we utilize RemoteCLIP/ViT L-14 as the teacher model due to its robust generalization capabilities in the remote sensing domain. The student model, CLIP/ViT B-32, serves as a lightweight counterpart to evaluate the efficiency of the proposed knowledge distillation process in transferring knowledge and capabilities from the larger teacher model. This setup follows the evaluation protocols of recent remote sensing CLIP baselines, enabling a fair comparison while isolating the contribution of our two-stage KD + RL refinement.

Reproducibility and variance: Unless otherwise stated, we report the primary metrics from a single run with a fixed random seed for each setting, consistent with common practice in recent CLIP-style remote sensing benchmarks and to keep the large-scale training budget tractable. Importantly, our “RL” stage is implemented as reward-shaped optimization on minibatches (no stochastic environment rollouts); the rewards are deterministic given the batch and current model parameters. Together with the decaying schedule w(t), this greatly stabilizes training compared to policy-gradient RL in interactive environments. We provide additional discussion of variance sources and practical mitigation (seed fixing, reward normalization, and early stopping) in Section 5.

### 4.2. Efficiency Analysis

We quantify efficiency in terms of (i) inference-time compute of the deployed student model and (ii) the extra training-time overhead introduced by Stage 2. Since the text prompts are fixed at inference, their embeddings are pre-computed and cached; therefore, online cost is dominated by the image encoder. Table 1 summarizes the parameter count and multiply–accumulate operations (MACs) of the teacher (ViT-L/14) and student (ViT-B/32) image encoders at 224×224 resolution, computed analytically from the backbone configuration.

**Training-time overhead.** Inference-time cost is unchanged because RLayer is only used during Stage 2 training and is removed at inference, while the student backbone remains ViT-B/32. The refinement stage adds a small training overhead: we run Stage 2 for 5 epochs by default, corresponding to roughly 10–20% of the total training steps in our experiments. Moreover, the per-step overhead of reward computation is negligible (<2%) since teacher cluster centers are pre-computed and the reward terms consist of lightweight cosine-similarity and probability lookups. Overall, KDRL incurs a modest additional training cost compared with standard KD/fine-tuning, while keeping inference efficiency identical to the student backbone.

The deployed KDRL model therefore retains the compact ViT-B/32 backbone at inference, yielding a 3.5× reduction in parameters and an 18× reduction in image-encoder MACs relative to the ViT-L/14 teacher. Stage 2 adds training cost but does not require expensive environment interaction: reward computation uses pre-computed teacher cluster centers and batch-level statistics, adding only lightweight operations on embeddings. As a result, KDRL targets edge-friendly inference while using a moderate, offline refinement budget during training.

### 4.3. Zero-Shot Classification on Remote Sensing Dataset

This subsection reports zero-shot remote sensing classification results to evaluate how well the distilled student generalizes without task-specific training. We first summarize the datasets and evaluation protocol (Section 4.3), then present the benchmark comparison and provide a critical interpretation of dataset-specific gains and degradations.

We consider widely used remote sensing benchmarks that vary in spatial resolution, geographic diversity, and scene semantics. This diversity is important for evaluating whether KDRL improves robustness under domain shift rather than overfitting to a single dataset style.

#### 4.3.1. Public Datasets for Image Classification

For completeness, we summarize additional remote sensing classification datasets used in subsequent supervised or ablation experiments, including their class counts, splits, and evaluation conventions.

To assess the effectiveness of the proposed KDRL framework for zero-shot classification, we conduct experiments on a variety of benchmark datasets widely used in the remote sensing domain: AID [29], EuroSAT [30], fMoW [31], Million-AID [32], PatternNet [33], NWPU-RESISC45 [34], and RSI-CB256 [35]. These datasets encompass a diverse range of scenes and environments, providing a rigorous evaluation ground for the generalization capabilities of the proposed framework. The datasets were curated, prepared, and made available through SkyScript [36], a comprehensive platform for remote sensing data processing and analysis. To ensure robust zero-shot performance, our model is fine-tuned on the SkyScript training dataset, which is specifically designed to enhance generalization across diverse classes and domains. Classification performance is quantified using accuracy, a standard metric that measures the proportion of correctly classified instances across all categories. The use of multiple datasets ensures a thorough evaluation, capturing the adaptability and scalability of KDRL for zero-shot tasks.

#### 4.3.2. Ablation Study

We conducted an ablation study to assess the individual and combined contributions of the key components of the KDRL framework. Specifically, we evaluated the impact of the supervised fine-tuning (SFT) stage, which includes logit matching, feature alignment, and task-specific loss, and the reinforcement-based refinement (ReFT) stage, which incorporates confidence-based rewards, cluster-based rewards, and dynamic KD loss decay. The results of this ablation study are summarized in Table 2.

As shown in Table 2, the baseline models demonstrate that CLIP with ViT L-14 achieves an accuracy of 61.30%, while ViT B-32 achieves 53.02%. These models serve as the baseline for comparison. When only logit matching is applied (KD logit), the student model’s accuracy improves to 55.98%, demonstrating the benefit of aligning logits. Similarly, applying feature alignment (KD feature) alone increases accuracy to 56.36%, showing that aligning intermediate features enhances performance. Combining both logit and feature alignment (KD full) results in a further accuracy improvement of 56.92%, highlighting the value of both components in refining the student model.

Introducing RL via confidence-based rewards (RL Confidence) results in a slight accuracy increase to 53.46%, suggesting that RL provides guidance for the student model. Adding cluster-based rewards (RL Cluster) yields a larger accuracy boost to 56.70%, indicating that aligning the features with the teacher’s cluster centers has a significant positive impact on performance. When both confidence-based and cluster-based rewards are combined (RL full), the accuracy reaches 56.93%, further refining the student model.

The use of dynamic KD with RL (Dynamic RL) leads to an accuracy of 57.34%, demonstrating the benefits of gradually transitioning from KD to RL to improve generalization. Finally, combining all components of the KDRL framework—logit matching, feature alignment, confidence-based reward, cluster-based reward, and dynamic KD loss decay—achieves the highest accuracy of 59.22%. This result underscores the effectiveness of the full KDRL framework in enhancing the student model’s performance through synergistic contributions from both KD and RL.

#### 4.3.3. Zero-Shot Classification Benchmark

Table 3 presents the zero-shot classification results on public datasets, with models trained on the SkyScript dataset. The benchmarks include CLIP-based methods, KD approaches, and the proposed KDRL method, which is also a CLIP-based approach built on RemoteCLIP. Performance is evaluated across datasets such as AID, EuroSAT, fMoW, Million-AID, PatternNet, RESISC, and RSI-CB.

Among the CLIP-based models, SkyCLIP-50 achieves high performance across most datasets, with notable results such as 80.88% on PatternNet and 67.45% on Million-AID. However, its performance is slightly lower on RESISC (70.94%) compared to RemoteCLIP (74.31%), showing variability depending on the dataset. RemoteCLIP, while strong on RSI-CB, struggles on more challenging datasets like fMoW (16.77%) and EuroSAT (27.81%).

KD-based methods consistently outperform standard CLIP-based approaches by leveraging efficient knowledge transfer. Among these, KD-TAKD achieves the best results, with scores of 48.01% on EuroSAT, 67.62% on Million-AID, and 78.41% on PatternNet. GDKD is a close second, excelling on PatternNet (78.43%) and Million-AID (67.77%). These methods demonstrate the power of KD in improving the generalization and efficiency of smaller models.

As a CLIP-based method derived from RemoteCLIP, KDRL combines KD with RL to optimize knowledge transfer and exploration. KDRL outperforms other methods on most datasets, achieving 69.51% on SkyScript, 69.24% on AID, 70.30% on RESISC, and 80.08% on PatternNet. On challenging datasets like fMoW, KDRL scores 20.31%, a significant improvement over RemoteCLIP’s 16.77%. These results highlight KDRL’s ability to overcome the limitations of RemoteCLIP and deliver robust performance across diverse datasets.

The results show that integrating reinforcement learning into knowledge distillation can significantly enhance zero-shot classification performance. KDRL not only addresses the weaknesses of RemoteCLIP but also surpasses both CLIP-based and KD-based methods across most datasets. Its dynamic optimization approach makes it highly effective in handling the spatial and temporal complexities of remote sensing data.

Compared with KD-TAKD, KDRL is slightly weaker on EuroSAT and fMoW. KD-TAKD introduces an intermediate teacher-assistant, which can reduce the capacity gap and preserve fine-grained teacher information in a more gradual manner. This can be advantageous when (i) the target dataset is relatively compact with high signal-to-noise (EuroSAT) or (ii) scenes exhibit strong context and temporal bias (fMoW). In contrast, our Stage 2 refinement emphasizes global representation geometry (cluster reward) and confidence shaping, which may slightly over-regularize the student toward teacher embedding clusters learned on the pre-training distribution. This trade-off favors generalization on most datasets but can be suboptimal on EuroSAT and fMoW, where dataset-specific cues are more prominent. We view this behavior as a limitation and discuss directions to mitigate it (e.g., task-adaptive cluster construction or reward weighting) in Section 5.

### 4.4. Image–Text Retrieval on Remote Sensing Dataset

This subsection evaluates whether the KDRL representation refinement transfers beyond classification to cross-modal image–text retrieval. Because retrieval depends on global embedding alignment between modalities, it provides a complementary view of how the student embedding geometry changes after Stage 2.

#### 4.4.1. Public Datasets for Image–Text Retrieval

We compare our KDRL against other baseline retrieval models, including VSE++ [42], SCAN [43], MTFN [44], AMFMN [45], LW-MRC [46], GaLR [47], CMFM-Net [48], HyperMatch [49], HVSA [50], FBCLM [51], DOVE [52], and PIR [53]. The evaluation is conducted by performing image-to-text and text-to-image retrieval tasks on two public datasets, RSITMD [54] and RSICD [55].

#### 4.4.2. Retrieval Benchmark

We report Recall@K (higher is better) for both image-to-text and text-to-image retrieval. Beyond absolute numbers, we analyze which gains are consistent across datasets and which failures suggest limitations of global reward shaping.

We conducted a comprehensive evaluation of our proposed KDRL method against state-of-the-art models on two public datasets, RSITMD and RSICD, for image-to-text and text-to-image retrieval tasks. Table 4 presents the comparative results in terms of Recall@1, Recall@5, and Recall@10.

For RSITMD, KDRL demonstrates substantial performance improvements, achieving 18.01% and 15.60% Recall@1 for image-to-text and text-to-image tasks, respectively, significantly outperforming most baseline methods. Notably, KDRL surpasses recent methods such as PIR and DOVE, particularly in Recall@10, with 67.44% and 74.76%.

On RSICD, KDRL continues to lead, with Recall@1 scores of 11.22% for text-to-image tasks. While FBCLM and PIR perform strongly in some metrics, KDRL establishes a robust advantage in Recall@10, reaching 52.61% and 57.04%, which are among the highest across all tested methods.

These results affirm the effectiveness of KDRL in bridging the gap between image and text modalities, achieving new state-of-the-art performance in key retrieval benchmarks.

### 4.5. Visual Grounding on Remote Sensing Dataset

This subsection evaluates KDRL on DIOR-RSVG visual grounding, which requires aligning a text query with a region (box) rather than only global embeddings. This task is therefore a stress test for whether Stage 2 rewards (defined on global embedding clusters and confidence) translate to spatially precise localization.

In this section we evaluate KDRL on a visual grounding task. We use Faster R-CNN as our RPN and replace the classification head with our CLIP model to perform evaluation on DIOR-RSVG dataset.

From Table 5, KDRL is less competitive under permissive thresholds (Pr@0.5–0.6) but becomes competitive under stricter thresholds (Pr@0.7–0.9), especially compared to other ViT-B/32 baselines.

Evaluation note: following the DIOR-RSVG protocol, Pr@τ reports the precision after filtering region–text matches by a similarity/confidence threshold τ∈{0.5,0.6,0.7,0.8,0.9}. Lower τ is more permissive (more predicted matches are kept), while higher τ focuses on the most confident alignments. At lower precision thresholds, KDRL demonstrates a notable performance gap, with RemoteCLIP achieving a 5.55% higher Pr@0.5 and SkyCLIP outperforming by 2.66%. Although the gap narrows at higher thresholds, such as Pr@0.9, where KDRL achieves comparable performance to SkyCLIP (64.21% vs. 62.11%), it still trails RemoteCLIP (63.38%). This trend suggests that while KDRL may perform adequately in stricter matching conditions, its ability to generalize under less restrictive thresholds is limited. This behavior is closely tied to our training objective and the DIOR-RSVG evaluation protocol. Stage 2 rewards are computed on global image–text embeddings and confidence, which tends to sharpen the similarity distribution for the top-ranked matches but does not explicitly optimize region-level representations or spatial alignment. Under permissive thresholds (τ=0.5 or 0.6), many medium-confidence region proposals are retained; without region-aware distillation, these proposals include more false positives, reducing precision. As τ increases, evaluation focuses on the most confident matches, and the improved global semantics from cluster-based refinement helps KDRL approach (or exceed) other ViT-B/32 baselines. The remaining gap to RemoteCLIP (L-14) is expected given the student’s smaller capacity and the absence of localization-aware rewards; improving grounding likely requires incorporating region-level cluster rewards or cross-attention/box supervision in Stage 2 rather than relying solely on global feature refinement.

The RL refinement stage explicitly optimizes (i) alignment to teacher cluster structure and (ii) prediction confidence. This tends to sharpen the score distribution: a small set of highly confident matches become more accurate, which improves Pr@0.7–0.9, while a larger set of moderately scored matches may remain poorly calibrated, which hurts Pr@0.5–0.6. In contrast, the larger RemoteCLIP/SkyCLIP teachers have higher capacity for region–text alignment under permissive filtering, leading to higher precision when many ambiguous proposals are retained. This analysis motivates future grounding-specific rewards (e.g., region localization cues) to better balance calibration across thresholds. While KDRL achieves competitive results at higher precision levels, further optimization is necessary to close the performance gap with other state-of-the-art CLIP-based models.

## 5. Discussion and Conclusions

**Figure 2:**
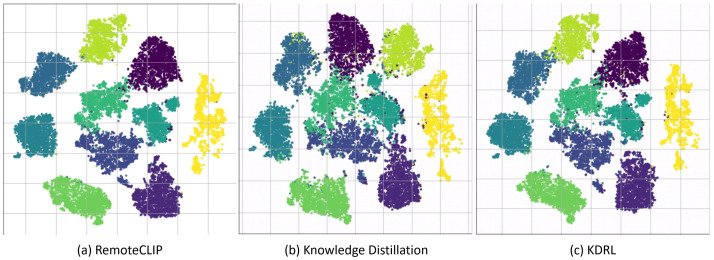
The feature clustering comparison between three different approaches (**a**) RemoteCLIP (teacher model), (**b**) knowledge distillation, and (**c**) KDRL illustrates the effectiveness of the KDRL method in refining the student model’s feature representation.

In addition to the experiments, we also visualize the feature clustering results to further analyze the performance of the models. Figure 2 presents a comparison of feature clustering between three approaches: RemoteCLIP (teacher model), KD, and KDRL. This comparison provides insights into the effectiveness of the KDRL framework.

In Figure 2a, the teacher model (RemoteCLIP) shows well-separated and compact clusters, indicating that the model produces highly discriminative feature representations for each class. These clusters represent the target feature space that the student model aims to replicate. On the other hand, Figure 2b, which shows the clustering results of the student model after applying standard KD, reveals that while the clusters remain distinct, there is noticeable misalignment and reduced compactness in the feature embeddings. This suggests that although KD aids in feature transfer, the alignment between the student and teacher features is less precise, and the compactness is not fully achieved.

In Figure 2c, we show the clustering results of our proposed KDRL approach. Compared to both the teacher model and the KD student, the clusters are more compact and better separated. This improvement in clustering quality indicates the contribution of reinforcement learning during the refinement phase. The additional rewards guide the student model to align its features more closely with the teacher’s cluster centers. The enhanced clustering results demonstrate that KDRL not only facilitates knowledge transfer through KD but also fine-tunes the feature space via RL technique, leading to better generalization across tasks.

### 5.1. Strengths and Weaknesses Across Tasks

Across tasks, KDRL is strongest when the downstream objective is dominated by global semantic alignment—zero-shot classification and image–text retrieval. In these settings, Stage 1 distillation transfers the teacher’s broad vision–language knowledge, while Stage 2 improves representation compactness (Figure 2) and reduces domain-specific feature drift, yielding consistent gains over the ViT-B/32 baselines (Table 3 and Table 4).

In particular, the proposed framework is most beneficial when strong global semantic transfer is required, while grounding-oriented extensions are needed for region-level tasks.

In contrast, the visual grounding task exposes a weakness: grounding requires fine-grained region–language correspondence and good score calibration over many ambiguous proposals. As shown in Table 5, KDRL improves precision in the high-confidence regime but remains weaker under permissive thresholds, suggesting that the current rewards are better aligned with global semantics than with dense localization.

### 5.2. Failure Modes

We qualitatively observe three typical failure patterns for the student model: (i) small or cluttered objects where the ViT-B/32 image encoder (larger patch size) lacks fine spatial detail; (ii) attribute confusion (e.g., visually similar land-use categories) where prompts contain subtle distinctions; and (iii) long-tail phrasing in text queries where limited domain-specific grounding supervision reduces robustness. These failure modes are most visible in grounding and, to a lesser extent, in retrieval under permissive filtering.

### 5.3. Why RL Helps Clustering but Can Hurt Grounding

The cluster reward explicitly encourages the student embedding to move toward teacher cluster centers, which directly improves intra-class compactness and inter-class separation—precisely what is visualized in Figure 2. However, grounding depends on ranking regions rather than whole-image embeddings. Because our rewards are computed on embedding alignment and confidence, they can over-emphasize global separability and “peakiness” of scores, which helps high-threshold precision but may reduce calibration when many candidate regions are retained (Table 5). This explains the apparent tension: RL improves the structure of the embedding space, yet does not directly optimize region-level localization.

### 5.4. Ablation Interpretation and Stability

Table 2 indicates that the cluster-based reward contributes more than the confidence reward. This is expected because the cluster term provides a structured, teacher-informed target for intermediate representations, while confidence alone can be satisfied by over-confident predictions without improving alignment. The dynamic schedule w(t) further mitigates instability by gradually shifting optimization from imitation (KD) to refinement (reward-shaped loss), reducing gradient conflict between task loss and reward terms. In practice, our “RL” stage is deterministic minibatch optimization rather than interactive rollouts, which helps control variance (see the reproducibility note in Section 4.2).

### 5.5. Computational Overhead and Deployment Implications

From a deployment perspective, KDRL is designed for edge inference: the final model keeps the compact ViT-B/32 student backbone, and text embeddings can be cached for fixed prompt sets. The additional Stage 2 refinement increases training time but does not add the heavy runtime cost of the teacher. This is particularly relevant for remote sensing platforms such as UAVs, satellites, and field-deployed systems where compute and memory budgets are limited, yet robust generalization is needed under domain shift (season, sensor, and geography).

### 5.6. Limitations and Future Work

KDRL has several limitations. First, although Stage 2 uses a deterministic and differentiable reward objective, a full multi-seed evaluation with standard deviations would strengthen the statistical claims since RL-style training objectives can still be sensitive to initialization and data shuffling. Second, our rewards are primarily designed for global semantic alignment; consequently, region-level localization is not explicitly optimized, which contributes to weaker visual grounding at permissive thresholds. Third, KDRL introduces additional hyperparameters (δ, η, λ, and the number of refinement epochs). While we observed stable defaults across benchmarks, these parameters can affect performance (especially grounding) and require validation tuning. Fourth, the two-stage pipeline increases training complexity compared with a single-stage fine-tuning or KD baseline, even though its additional wall-clock cost is modest (Section 4.2).

Future work will explore (i) automating reward design and weighting (e.g., via meta-learning or adaptive schedules); (ii) incorporating localization-aware and calibration-aware rewards (e.g., region-level cluster rewards, cross-attention supervision, and temperature scaling); (iii) extending KDRL to online/continual learning settings where clusters and rewards update as data streams; and (iv) investigating alternative RL algorithms (e.g., actor–critic or off-policy methods) for the refinement stage.

This work demonstrates the potential of combining knowledge distillation and reinforcement learning to enhance model performance, particularly in domains with complex data distributions. The proposed KDRL framework achieves superior feature alignment, improved generalization, and task performance, setting a new standard for lightweight model training in image processing tasks.

## Figures and Tables

**Table 1:** Teacher vs. student model footprint (image encoder only, 224×224). *N* is the number of tokens including the class token. MACs are reported per forward pass; smaller is better.

Model	*N*	Params (M)	MACs (G)
Teacher: ViT-L/14	257	303.7	81.1
Student: ViT-B/32	50	87.8	4.4

**Table 2:** Ablation study evaluating the contributions of individual components in the proposed framework. The table compares various configurations of KD and RL techniques. The model configurations are tested on CLIP variants (ViT L-14 and ViT B-32), showing the impact of different methods on overall accuracy. Here, BB denotes the backbone architecture.

Model			KD		RL		Accuracy
Name	BB	Method	Logit	Feature	Confidence	Cluster	
CLIP	ViT L-14	SFT	-	-	-	-	61.30%
CLIP	ViT B-32	SFT	-	-	-	-	53.02%
KD logit			✓	-	-	-	55.98%
KD feature	ViT B-32	SFT	-	✓	-	-	56.36%
KD full			✓	✓	-	-	56.92%
RL Confidence			-	-	✓	-	53.46%
RL Cluster	ViT B-32	ReFT	-	-	-	✓	56.70%
RL full			-	-	✓	✓	56.93%
Dynamic RL	ViT B-32	Dynamic	-	-	✓	✓	57.34%
KDRL	ViT B-32	Dynamic	✓	✓	✓	✓	59.22%

**Table 3:** Benchmark evaluation on zero-shot classification on public datasets. The methods are trained on the SkyScript dataset.

ViT	Model	SkyScript	Zero-Shot Classification						
			AID	EuroSAT	fMoW	Million-AID	PatternNet	RESISC	RSI-CB
L-14	CLIP-original [37]	55.06	69.25	41.89	26.19	57.88	71.39	66.70	43.02
	RemoteCLIP [38]	34.40	70.85	27.81	16.77	47.20	61.91	74.31	50.79
	SkyCLIP-50 [36]	70.89	71.70	51.33	27.12	67.45	80.88	70.94	50.09
B-32	CLIP-original [37]	40.16	69.55	32.11	17.62	57.27	64.09	65.71	41.26
	RemoteCLIP [38]	29.06	68.05	30.74	11.13	46.26	56.05	67.88	46.55
	CLIP-caption [36]	40.03	71.05	33.85	18.02	57.48	66.56	66.04	42.73
	CLIP-laion-RS [36]	40.77	69.55	37.63	19.16	56.59	64.79	64.63	41.79
	SkyCLIP-50 [36]	52.98	70.90	33.30	19.24	62.69	72.18	66.67	46.20
B-32	KD-Logit [1]	64.20	67.72	44.19	18.44	63.59	76.98	66.70	46.05
	KD-Feature [39]	64.71	66.98	44.33	19.41	63.80	77.29	68.51	45.85
	KD-TAKD [40]	69.07	68.08	48.01	20.64	67.62	78.41	68.40	46.28
	GDKD [41]	67.56	67.58	46.89	20.23	67.77	78.43	67.97	46.59
B-32	KDRL	69.51	69.24	47.92	20.31	67.55	80.08	70.30	48.85

**Table 4:** Benchmark evaluation of cross-modal image and text retrieval on RSITMD and RSICD datasets.

Method	RSITMD						RSICD					
	Image to Text			Text to Image			Image to Text			Text to Image		
	R@1	R@5	R@10	R@1	R@5	R@10	R@1	R@5	R@10	R@1	R@5	R@10
VSE++ [42]	10.38	27.65	39.60	7.79	24.87	38.67	3.38	9.51	17.46	2.82	11.32	18.10
SCAN t2i [43]	10.18	28.53	38.49	10.10	28.98	43.53	4.39	10.90	17.64	3.91	16.20	26.49
SCAN i2t [43]	11.06	25.88	39.38	9.82	29.38	42.12	5.85	12.89	19.84	3.71	16.40	26.73
MTFN [44]	10.40	27.65	36.28	9.96	31.37	45.84	5.02	12.52	19.74	4.90	17.17	29.49
AMFMN-soft [45]	11.06	25.88	39.82	9.82	33.94	51.90	5.05	14.53	21.57	5.05	19.74	31.04
AMFMN-fusion [45]	11.06	29.20	38.72	9.96	34.03	52.96	5.39	15.08	23.40	4.90	18.28	31.44
AMFMN-sim [45]	10.63	24.78	41.81	11.51	34.69	54.87	5.21	14.72	21.57	4.08	17.00	30.60
LW-MRC-u [46]	9.73	26.77	37.61	9.25	34.07	54.03	4.39	13.35	20.29	4.30	18.85	32.34
GaLR [47]	14.82	31.64	42.48	11.15	36.68	51.68	6.59	19.85	31.04	4.69	19.48	32.13
CMFM-Net [48]	10.83	28.76	40.04	10.00	32.83	47.21	5.40	18.66	28.55	5.31	18.57	30.03
HyperMatch [49]	11.73	28.10	38.05	9.16	32.31	46.64	7.14	20.04	31.02	6.08	20.37	33.82
HVSA [50]	13.20	32.08	45.58	11.43	39.20	57.45	7.47	20.62	32.11	5.51	21.13	34.13
FBCLM [51]	12.84	30.53	45.89	10.44	37.01	57.94	13.27	27.17	37.60	13.54	38.74	56.94
DOVE [52]	16.81	36.80	49.93	12.20	49.93	66.50	8.66	22.35	34.95	6.04	23.95	40.35
PIR [53]	18.14	41.15	52.88	12.17	41.68	63.41	9.88	27.26	39.16	6.97	24.56	38.92
KDRL	18.01	49.52	67.44	15.60	56.17	74.76	12.47	37.92	52.61	11.22	37.24	57.04

**Table 5:** Performance of our KDRL on visual grounding.

Method	DIOR-RSVG				
	Pr@0.5	Pr@0.6	Pr@0.7	Pr@0.8	Pr@0.9
RemoteCLIP (L-14)	33.62	51.29	61.81	65.61	72.22
SkyCLIP (L-14)	31.98	48.01	57.30	63.77	69.38
RemoteCLIP (B-32)	31.53	45.71	54.35	60.02	63.38
SkyCLIP (B-32)	28.64	42.86	54.03	59.32	62.11
KDRL (B-32)	25.98	43.10	55.53	60.84	64.21

## Data Availability

The experimental data used in this study are derived entirely from publicly available remote sensing image–text datasets. Specifically, we use the RSICD dataset, available at 1 December 2025 https://github.com/201528014227051/RSICD_optimal, and the RSITMD dataset, available at 1 December 2025 https://github.com/AICyberTeam/AMFMN/tree/main/RSITMD.
